# Activating Somatic *FGFR2* Mutations in Breast Cancer

**DOI:** 10.1371/journal.pone.0060264

**Published:** 2013-03-20

**Authors:** Nadine Reintjes, Yun Li, Alexandra Becker, Edyta Rohmann, Rita Schmutzler, Bernd Wollnik

**Affiliations:** 1 Institute of Human Genetics, University of Cologne, Cologne, Germany; 2 Center for Molecular Medicine Cologne (CMMC), University of Cologne, Cologne, Germany; 3 Cologne Excellence Cluster on Cellular Stress Responses in Aging-Associated Diseases (CECAD), University of Cologne, Cologne, Germany; 4 Department of Molecular Gynecology and Oncology, Gynecology and Obstetrics Clinic, University of Cologne, Cologne, Germany; Virginia Commonwealth University, United States of America

## Abstract

It is known that *FGFR2* gene variations confer a risk for breast cancer. FGFR2 and FGF10, the main ligand of FGFR2, are both overexpressed in 5–10% of breast tumors. In our study, we sequenced the most important coding regions of *FGFR2* in somatic tumor tissue of 140 sporadic breast cancer patients and performed MLPA analysis to detect copy number variations in *FGFR2* and *FGF10*. We identified one somatic heterozygous missense mutation, p.K660N (c.1980G>C), within the tyrosine kinase domain of FGFR2 in tumor tissue of a sporadic breast cancer patient, which is likely mediated by the FGFR2-IIIb isoform. The presence of wild type and mutated alleles in equal quantities suggests that the mutation has driven clonal amplification of mutant cells. We have analyzed the tyrosine kinase activity of p.K660N and another recently described somatic breast cancer mutation in FGFR2, p.R203C, after expression in HEK293 cells and demonstrated that the intrinsic tyrosine kinase activity of both mutant proteins is strongly increased resulting in elevated phosphorylation and activity of downstream effectors. To our knowledge, this is the first report of functional analysis of somatic breast cancer mutations in FGFR2 providing evidence for the activating nature of FGFR2-mediated signalling in the pathogenesis of breast cancer.

## Introduction

Genome-wide association studies identified variants in the fibroblast growth factor receptor 2 (*FGFR2*) tumor suppressor gene as a genetic risk factor for breast cancer susceptibility. The most strongly associated variants were located in intron 2 of the *FGFR2* gene [1], [2]. Intron 2 of *FGFR2* shows a high degree of conservation in mammals and contains several putative transcription factor binding sites, some of which lie in close proximity to the relevant variants. Therefore, it is speculated that the association with breast cancer is mediated through the regulation of *FGFR2* expression [2]. The expression and amplification of FGFR2 has long been known to be elevated in 5–10% of breast tumors [3]. Somatic missense mutations of *FGFR2* that are likely to be implicated in cancer development have also been demonstrated in primary tumors and cell lines of multiple tumor types [4], [5].

Other genome-wide association studies of breast cancer predisposition identified variants on chromosome 5p12 some 274–317 kb distal to the fibroblast growth factor 10 (*FGF10*) that confer a risk, preferentially for estrogen receptor-positive breast tumors. *FGF10* is amplified in approximately 10% of breast cancers, and possibly the observed risk variants influence *FGF10* expression [6].

Mouse models of mammary carcinogenesis have long established the FGF signalling pathway as a major contributor to tumorigenesis [7], and a mouse mammary tumor virus (MMTV) insertional mutagenesis screen for genes involved in breast cancer has identified both *FGFR2* and *FGF10* [8]. However, as yet, little is known about the mechanism by which *FGFR2* and *FGF10* mutations act as risk factors in predisposition to breast cancer [9].

The receptor tyrosine kinase FGFR2 is one of four fibroblast growth factor receptors designated FGFR1-4 that activate FGF signalling upon trans-autophosphorylation of the receptor dimers. The activation of receptor tyrosine kinase signalling is one of the mechanisms underlying tumor development and growth.

The FGF system consists of at least 22 distinct FGFs which have been identified in a variety of organisms from nematode and drosophila to mouse and human. Although FGFs vary in size from 17 to 34 kDa, all members of the family share a conserved sequence of 120 amino acids that show 16–65% sequence identity [10]. The binding of FGFs to FGFRs in the presence of heparin sulphate glycosaminglycan induces receptor dimerization and activation of the protein tyrosine kinase domain [11]. Tyrosine autophosphorylation and the recruitment of a complement of downstream signalling molecules result in the stimulation of various signalling cascades that play critical roles in various cellular processes [12]. FGFs fulfill versatile functions throughout the human life cycle commencing at germ cell maturation [13], [14], continuing throughout embryonic development [15], [16], [17] and into adulthood [18]. During embryogenesis, FGFs are essential in morphogenesis by regulating cell proliferation, differentiation and cell migration [19], [20]. In the adult, FGFs continue to regulate tissue homeostasis but are also involved in the control of the nervous system, in tissue repair, wound healing, cholesterol metabolism [21], serum phosphate regulation [22] and tumor angiogenesis [12].

FGFRs share 55% to 72% homology at the protein level. Like all receptor tyrosine kinases, FGFRs are composed of an extracellular ligand binding domain, a transmembrane region, and a cytoplasmic region containing a catalytic protein tyrosine kinase core and additional regulatory sequences. The extracellular domain is composed of three immunoglobulin-like domains (designated D1–D3), a stretch of negatively charged amino acids in the linker connecting D1 and D2, termed the acidic box, and a conserved positively charged region in D2 that serves as the binding site for heparin sulphate or heparin [11], [23], [24], [25].

FGF-FGFR specificity is an essential mechanism in the regulation of FGF response and is achieved primarily through alternative splicing in the second half of D3 in FGFRs. Transcripts of FGFR1, 2, and 3, but not of FGFR4 are subject to alternative RNA splicing in which exon 7 of the *FGFR* gene encodes a common N-terminal half of D3 (referred to as IIIa) and two different exons 8 code for the C-terminal half of D3 to generate the IIIb and IIIc isoforms, respectively [26], [27]. The IIIb isoforms are expressed exclusively in epithelial cells, while the IIIc isoforms are expressed only in mesenchymal cells [28], [29], [30], [31]. Moreover, the IIIb and IIIc isoforms of FGFR1, -2 and -3 bind to different complements of FGFs that are expressed exclusively in mesenchymal or epithelial cells, respectively. Mammary epithelial cells express FGFR2-IIIb, which binds FGF7 and FGF10, expressed by surrounding mesenchymal cells.

We hypothesized that specific private mutations of an activating nature in *FGFR2* and *FGF10* might influence breast tumor development and growth. To test this hypothesis, we screened sporadic breast cancer patients for mutations in both candidate genes and further investigated identified breast cancer mutations by functional analysis.

## Materials and Methods

### Patients

DNA isolated from somatic tumor tissue of 140 sporadic breast cancer patients was collected by the Department of Molecular Gynecology and Oncology, Gynecology and Obstetrics Clinic, Cologne, Germany. Once a mutation was identified, DNA from non-tumor tissue and blood-derived DNA from the patient as well as blood-derived DNA from 200 control individuals was tested. Informed written consent for genetic analysis of samples was given by all subjects. Ethical approval for this study was given by the institutional Ethics Committee of the University of Cologne, Germany (07–185, 10/18/2007).

### Mutation analysis

The protein-coding exons 5, 7–9 and 12–15 of the IIIb isoform (NM_022970.3) and also exon 8 of the IIIc isoform (NM_000141.1) as well as adjacent intronic sequences of the *FGFR2* gene were amplified by PCR using standard conditions (Table S1). PCR fragments were purified and directly sequenced utilizing the corresponding forward or reverse primers with the ABI BigDye Terminator v3.1 Cycle Sequencing Kit and an ABI 3730 DNA Analyzer (Applied Biosystems by Life Technologies, Darmstadt, Germany). The identified mutation was resequenced in independent experiments. Primer design and DNA mutation numbering was given based on cDNA sequence of *FGFR2* GenBank entry NM_022970.3 with 1 corresponding to the A of the ATG translation initiation codon.

### Multiplex ligation-dependent probe amplification (MLPA) assay

The MLPA kit for the *FGF10* and *FGFR2* genes (SALSA P231) was purchased from MRC Holland (Amsterdam, The Netherlands). The probe mix contains 34 probes: 6 hybridizing to the *FGF10* gene, 8 to the *FGFR2* gene, and, as controls, 18 to single-copy genes located on other human chromosomes. The assay was conducted according to the instructions by the manufacturer. For the reactions, 100 ng DNA isolated from somatic tumor tissue was used. Amplification products were separated on an ABI PRISM 3100 Avant Genetic Analyzer (Applied Biosystems by Life Technologies, Darmstadt, Germany) using ROX 500 internal size standard (Serac, Bad Homburg, Germany). Data were retrieved by the GeneScan Software 3.7 (Applied Biosystems by Life Technologies, Darmstadt, Germany) and fragment sizes, peak areas and peak heights were determined with Sequence Pilot^CE^ software. Variations in peak area were evaluated by comparison of each sample with four controls always from the same experiment. Deletions were suspected when the peak area was lower than 75% of the controls, duplications were suspected when the peak area was higher than 125% of the controls, and multiple copies were suspected when the peak area was higher than 175% of the controls.

### FGFR2 isoform study

RNA was isolated from somatic tumor tissue of 3 sporadic breast cancer patients. First strand cDNA synthesis was carried out by the use of the RevertAid First Strand cDNA Synthesis Kit (Fermentas, St. Leon-Rot, Germany). Reverse transcription reaction was performed with oligo(dT)_18_ primer at 37°C for 1 h, followed by 72°C for 10 min. To examine the presence of FGFR2-IIIb and FGFR2-IIIc transcript isoforms in the three cDNA samples, a primer pair complementary to the cDNA sequence and flanking the alternative spliced exon 8 was designed (5′CATCGCTGATTCGCACATGACGGGCTGCC CTACCTCAAGG located in exon 7 and 3′GACTGTTACCACCATACAGG located in exon 9). The 5′primer was labeled with the fluorescence Hex and PCR products were resolved with an ABI PRISM 3100 Avant Genetic Analyzer (Applied Biosystems by Life Technologies, Darmstadt, Germany) using ROX 500 internal size standard (Serac, Bad Homburg, Germany). PCR product sizes were determined with GeneScan Software 3.7. Expected size of PCR product including exon 8 of the IIIb isoform was 297 bp and of PCR product including exon 8 of the IIIc isoform was 294 bp. Furthermore, PCR fragments were purified and directly sequenced utilizing the 5′ and 3′ primer with the ABI BigDye Terminator v3.1 Cycle Sequencing Kit and an ABI PRISM 3100 Avant Genetic Analyzer (Applied Biosystems by Life Technologies, Darmstadt, Germany).

### Generation of site-directed mutants

pRK5 expression vector, kindly provided by Joseph Schlessinger (Department of Pharmacology, Yale University School of Medicine, New Haven, Connecticut), was used for human wild-type (wt) and mutant FGFR2-IIIb transient expression in HEK293 cells. Point mutations in *FGFR2* were generated using a QuickChange site-directed mutagenesis kit (Agilent Technologies, Waldbronn, Germany).

### Cell culture and transfection

HEK293 cells were cultured in Dulbecco's Modified Eagle Medium (DMEM) containing 10% fetal bovine serum (FBS), 0.7 µg/ml amphothericin B, 100 U/ml penicillin and 100 µg/ml streptomycin at 37.8°C in an 5% CO_2_/95% air environment.

HEK293 cells were transiently transfected with Lipofectamine 2000 (Invitrogen by Life Technologies, Darmstadt, Germany) and incubated in transfection medium for 6 h. This was followed by changing the medium to DMEM containing 10% FBS. After 18 h, cells were washed with PBS and lysed for 5 min on ice in 50 mM HEPES, 150 mM NaCl, 1% TX-100, 10% glycerine plus protease inhibitors.

Additionally, HEK293 cells were transiently transfected using calcium phosphate. This was followed by changing the medium to DMEM devoid of FBS. After starvation overnight, cells were stimulated for 5 min with 25 ng/ml of growth factor FGF1 (R&D Systems, Wiesbaden Nordenstadt, Germany). FGF1 was prepared and used as a stock solution at a concentration of 100 µg/ml with 5 mg/ml heparin. Cells were washed with PBS and lysed for 5 min on ice in 150 mM NaCl, 0,5 mM EDTA, 1 % NP40, 20 mM Tris, 1 mM NaVO_4_, 10 mM NaF, 10 µM NaMO_4_ and proteinase inhibitors.

Lysates were centrifuged at 16100 g for 10 min at 4°C. The supernatant was designated the soluble fraction. Protein concentrations of soluble cellular fractions were determined using the BCA protein assay kit (Thermo Fisher Scientific, Bonn, Germany).

### Immunoprecipitation and immunoblot analysis of protein extracts

Proteins expressed in HEK293 cells transiently transfected with lifpofectamine were immunoprecipitated from soluble lysates using protein A/G PLUS agarose and antibody Bek (C-17), rabbit polyclonal IgG (Santa Cruz Biotechnology, Heidelberg, Germany) according to manufacturer's guidelines. Immunoprecipitates were washed in 20 mM HEPES, 150 mM NaCl, 0.1% TX-100 and 5% glycerine. Equal amounts of protein extracts were prepared by addition of NuPAGE LDS Sample Buffer and NuPAGE Sample Reducing Agent and heated at 95°C for 5 min. Samples were loaded on 7% NuPAGE Tris-Acetate Gels (Invitrogen by Life Technologies, Darmstadt, Germany). Proteins were transferred to nitrocellulose membranes by electrophoresis over night at 12 V at 4°C in NuPAGE Transfer Buffer (Invitrogen by Life Technologies, Darmstadt, Germany). Membranes were blocked with 5% non-fat dry milk in PBS for 1 h at room temperature (RT). Samples of cells transiently transfected with calcium phosphate, also previously prepared by addition of NuPAGE LDS Sample Buffer and NuPAGE Sample Reducing Agent and heated at 95°C for 5 min, were loaded on 4–12% NuPAGE Bis-Tris Gels (Invitrogen by Life Technologies, Darmstadt, Germany). Proteins were transferred to nitrocellulose membranes by electrophoresis for 3 h at 30 V at RT in NuPAGE Transfer Buffer and subsequently membranes were blocked with 5% non-fat dry milk in TBST buffer (TBST buffer: 0.1% Tween 20, 20 mM Tris, 150 mM NaCl) for 1 h at RT. After blocking, all membranes were incubated overnight at 4°C in TBST buffer containing the primary antibodies Bek (C-17) (Santa Cruz Biotechnology, Heidelberg, Germany), p-Tyr (PY99) (Santa Cruz Biotechnology, Heidelberg, Germany), p-FRS2-α (Tyr196) (Cell Signaling Technology by New England Biolabs GmbH, Frankfurt am Main, Germany), p-STAT3 (9E12) (Santa Cruz Biotechnology, Heidelberg, Germany), p-MEK1/2 (S217/221) (Cell Signaling) and Actin (Sigma-Aldrich, München, Germany). This was followed by washing the membranes with TBST buffer and an incubation for 1 h at 4°C with TBST buffer containing the secondary antibodies goat anti-rabbit IgG or goat anti-mouse IgG, conjugated to horseradish peroxidase (Santa Cruz Biotechnology, Heidelberg, Germany). Reactions were revealed after washing with SuperSignal West Pico Chemoluminescent Substrate (Thermo Fisher Scientific, Bonn, Germany).

## Results

### Identification of a heterozygous *FGFR2* mutation

**Figure 1:**
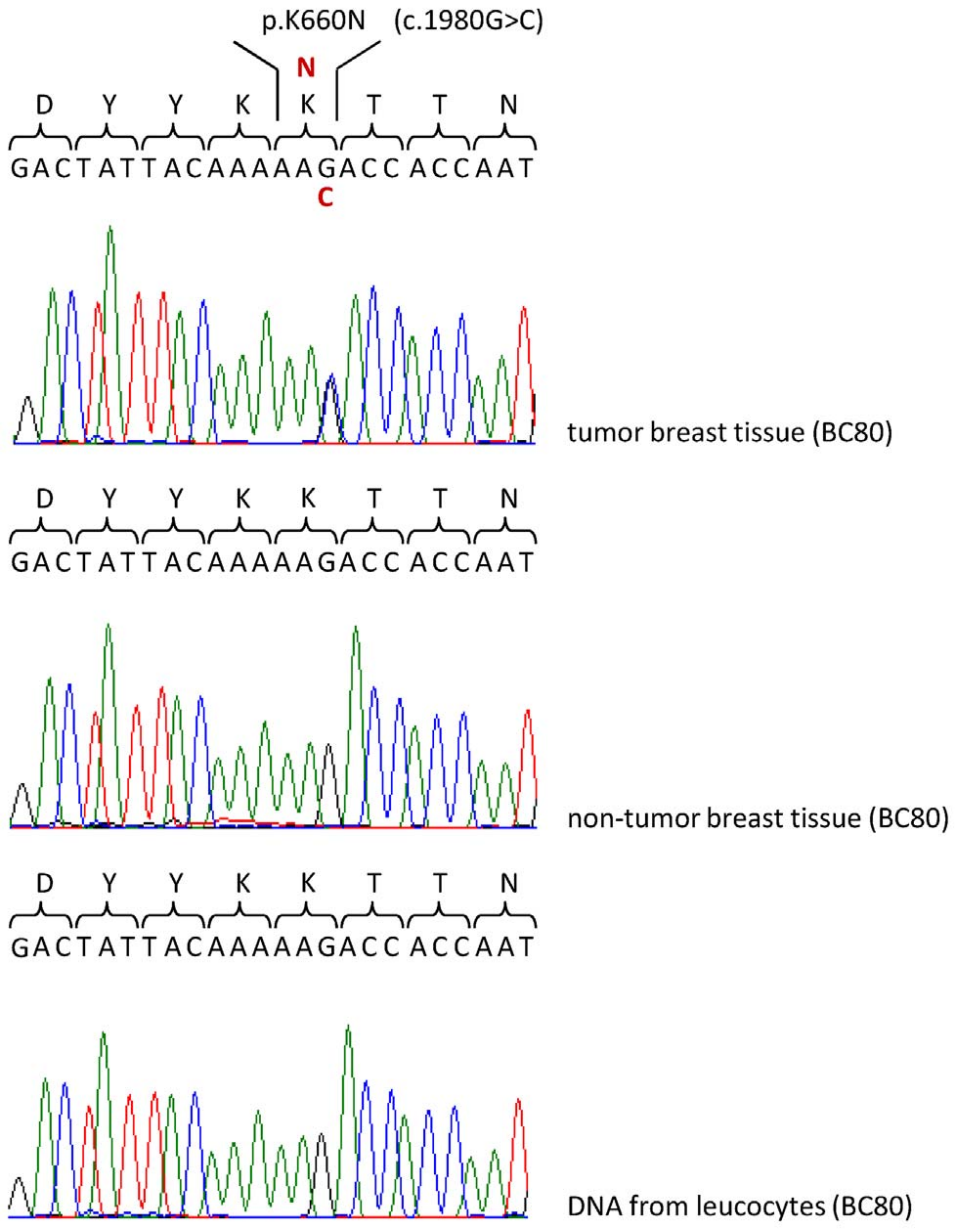
Identified *FGFR2* mutation in tumor tissue. The upper sequence chromatogram shows the heterozygous missense mutation in exon 14 in *FGFR2*, c.1980G>C (p.K660N), found in somatic tumor breast tissue of patient BC80. The middle and lower chromatograms illustrate the normal sequence in non-tumor breast tissue and blood-derived DNA of the same patient.

**Figure 2:**
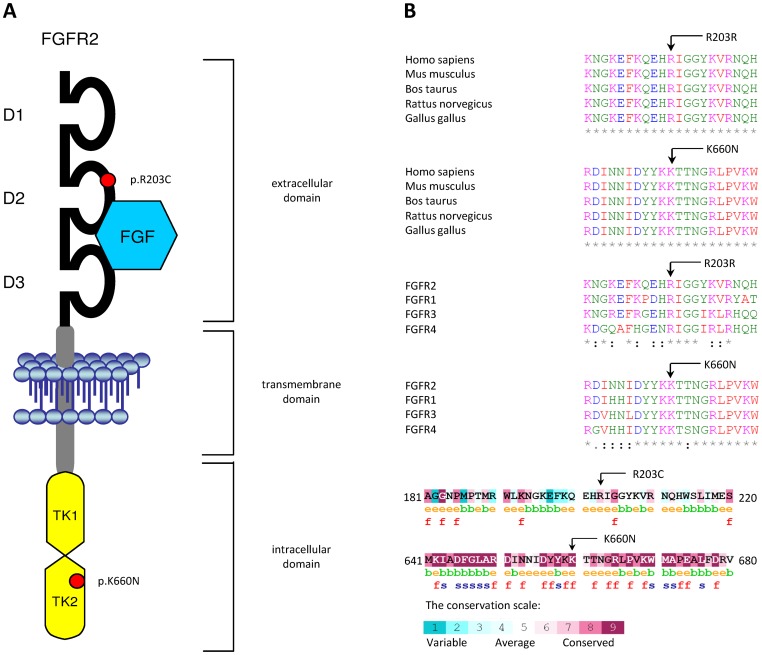
Location and conservation of FGFR2 mutations. A) Schematic model of FGFR2 with bound ligand (FGF). The locations of the novel p.K660N and p.R203C mutations are marked by red dots. TK1/2: tyrosine kinase domains 1 and 2; D1–3: immunoglobulin-like domains 1–3. **B) Conservation of FGFR2 mutations.** Arrows indicate localization of mutations. *Above* CLUSTALW alignment of vertebrate FGFR2s and human FGFRs. *Below*: ConSeq prediction. Amino acid conservation grade is colour-coded. The predicted status of each residue, buried (b) or exposed (e), is marked below the amino acid sequence. Slowly evolving and exposed residues are predicted to be functional (f), whereas slowly evolving and buried residues are predicted to be structurally important (s).

In our study we examined the immunoglobulin-like domains D2 and D3, the transmembrane domain and the tyrosine kinase domain of FGFR2, i.e. regions in which the typical mutational hot spots of craniosynostosis syndromes are located, in somatic tumor tissue of sporadic breast cancer patients. We sequenced the protein-coding exons 5 and 7–9 in about 50 patients and exons 12–15 in about 140 patients as well as adjacent intronic sequences and we identified one novel heterozygous missense mutation in exon 14 of *FGFR2*, c.1980G>C, in tumor tissue of one of them (BC80). This mutation is predicted to lead to a substitution of the lysine at position 660 to asparagine (p.K660N). The mutation was present neither in non-tumor tissue of the same patient nor in blood-derived DNA from the patient or 200 control individuals. In addition, the mutation has not been reported in the Human Gene Mutation Database (HGMD) (URL: http://www.hgmd.cf.ac.uk/ac/index.php) or the Exome Variant Server, NHLBI GO Exome Sequencing Project (ESP) (URL: http://evs.gs.washington.edu/EVS/). Considering the presence of wt and mutated alleles in equal quantities, the mutation must have either occurred at a very early stage of breast cancer development or expanded via clonal amplification of mutant cells (Fig. 1). Moreover, the lysine at position 660 is strongly conserved between species and all four human FGFRs, indicating that this site is of functional importance. Notably, inspection of the structure of the receptor showed that the breast cancer mutation is located in the intracellular tyrosine kinase domain adjacent to the activation loop suggesting that it might affect the tyrosine kinase activity of FGFR2 (Fig. 2).

In our patient cohort, we detected several single nucleotide polymorphisms, and allele frequencies were consistent with stored frequencies in the HapMap database (www.hapmap.org) (Table S2).

**Figure 3:**
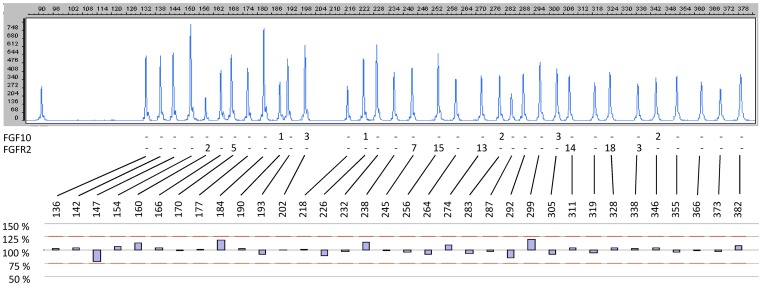
*FGFR2/FGF10* MLPA analysis. Representative MLPA chromatogram and quantification with SequencePilot software. The probe mix contained 34 probes, 6 hybridizing to the *FGF10* gene, 8 to the *FGFR2* gene, and 20 controls hybridizing to single-copy genes located on other chromosomes. Peak areas lower than 75% of the controls are indicative for a deletion, peak area higher than 125% of the controls for a duplication.

We also performed MLPA analysis to detect copy number variations in *FGFR2* and *FGF10* in breast tissue of 50 sporadic breast cancer patients. However, we did not identify any copy number changes in either of the two genes (Fig. 3).

### The FGFR2-IIIb isoform is predominantly expressed in tumor tissue of sporadic breast cancer patients

**Figure 4:**
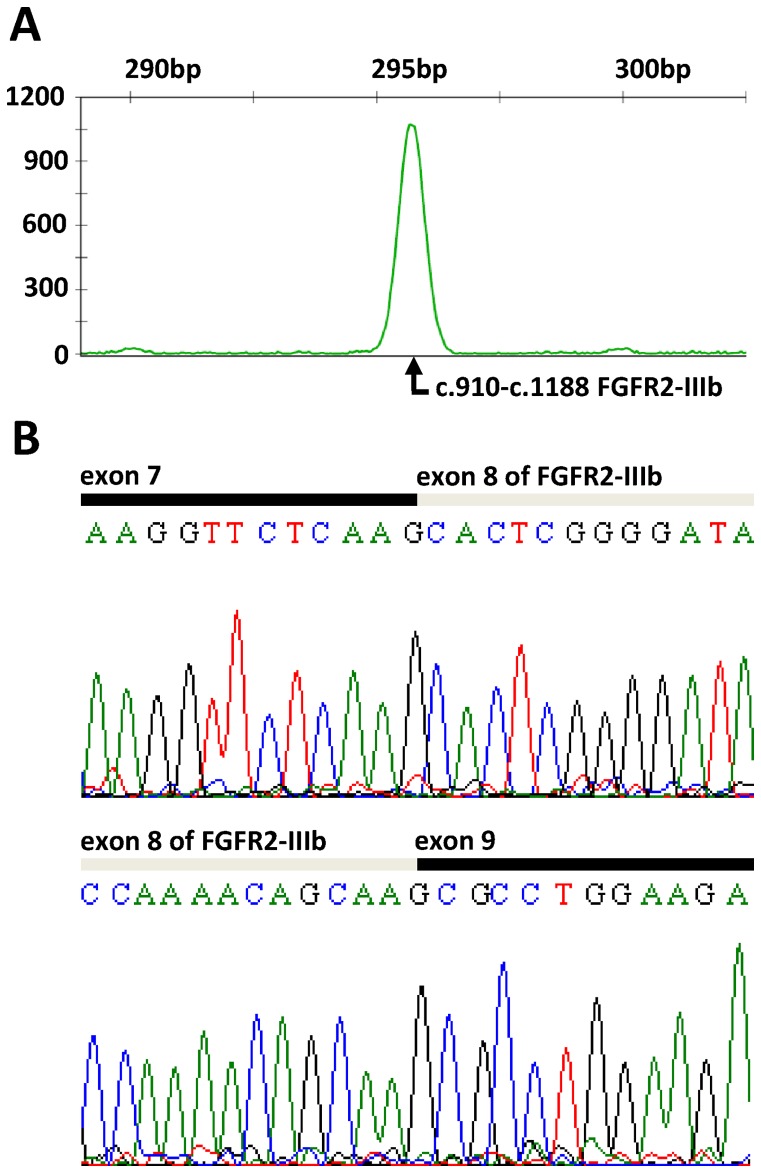
*FGFR2* mRNA isoform expression analysis in tumor tissue of three sporadic breast cancer patients. A) Representative electropherogram of GeneScan analysis. Isoforms FGFR2-IIIb and FGFR2-IIIc differ in exon 8, resulting in a variation of 3 bp in length of mature mRNA. A PCR fragment of 297 bp for *FGFR2*-IIIb or 294 bp for *FGFR2*-IIIc cDNA spanning exon 8 of both isoforms was amplified by PCR using a fluorescently-labeled primer pair located in exons 7 and 9, which are common in both isoforms. Fragment analysis showing a single sharp peak. **B) Representative sequence electropherogram showing the expression of IIIb isoform.**

The p.K660N mutation is located in a region common to both FGFR2-IIIb and FGFR2-IIIc isoforms. To determine whether the identified breast cancer mutation is primarily mediated by the FGFR2-IIIb or FGFR2-IIIc isoform, we examined the presence of the IIIb and IIIc transcripts in three cDNA samples synthesized from tumor tissue total RNAs of sporadic breast cancer patients. We designed a fluorescently labeled primer pair complementary to the cDNA sequence and flanking the alternative spliced exon 8. Primers were located in exons 7 and 9, which are common in the IIIb and IIIc transcripts, allowing amplification of exon 8 of both isoforms and the adjacent exon boundaries. The PCR product of isoform IIIb was expected to be three base pairs bigger than that of isoform IIIc. Amplification products were evaluated by GeneScan analysis. Notably, the electropherogram of the GeneScan analysis showed a single sharp peak in the case of all three tested cDNA samples suggesting the amplification of only one isoform (Fig. 4A). Subsequent direct sequencing of the products verified that predominantly the transcript of FGFR2-IIIb is present in tumor tissue of sporadic breast cancer patients (Fig. 4B).

### Increased tyrosine kinase activity of FGFR2 breast cancer mutant

**Figure 5:**
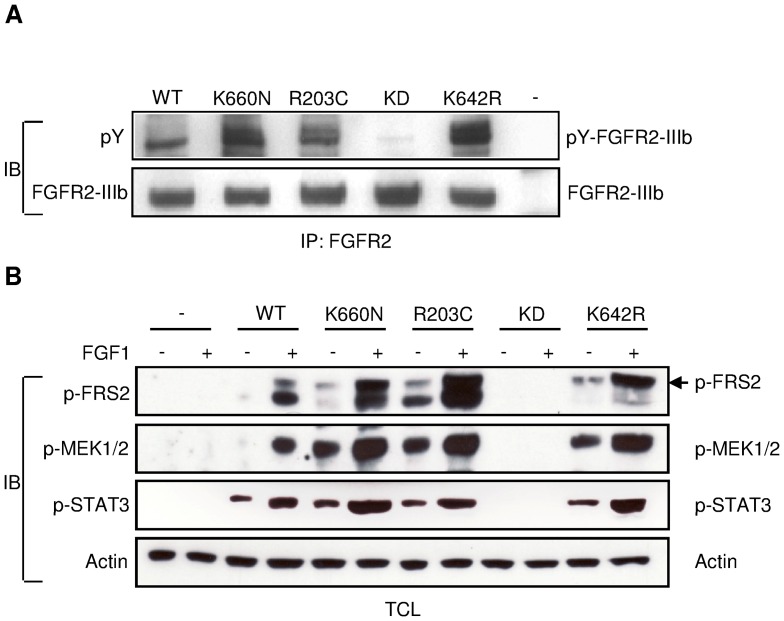
Activating FGFR2 breast cancer mutations. **A)** Representative Western blot showing increased tyrosine kinase activities of FGFR2-IIIb breast cancer mutants compared to tyrosine kinase activities of wt FGFR2-IIIb (WT), of a kinase defective (KD) FGFR2-IIIb mutant, and of a Pfeiffer syndrome gain-of-function mutant (K642R, previously named K641R according to another reference sequence) as controls. HEK293 cells were transiently transfected using lipofectamine with pRK5 vectors containing cDNA coding for wt FGFR2-IIIb or FGFR2-IIIb variants containing the indicated amino acid substitution. Untransfected cells served as negative control (−). Lysates from cells were subjected to immunoprecipitation with anti-FGFR2 antibodies (Bek(C-17)) followed by SDS-PAGE and immunoblotting (IB) with anti-FGFR2 or anti-p-Tyr antibodies (PY99). **B**) Representative Western blots showing increased substrate phosphorylation by FGFR2-IIIb breast cancer mutants compared to substrate phosphorylation by wt FGFR2-IIIb (WT), a kinase defective (KD) FGFR2-IIIb mutant, and a Pfeiffer syndrome gain-of-function mutant (K642R, previously named K641R according to another reference sequence) as controls. For transient transfection calcium phosphate was used. Untransfected cells served as negative control (−). HEK293 cells expressing FGFR2-IIIb as well as untransfected cells were stimulated with FGF1. Lysates of unstimulated or FGF1-stimulated cells were subjected to SDS-PAGE followed by immunoblotting with anti-p-FRS2, anti-p-MEK1/2, anti-p-STAT3 and anti-ß-Actin as loading control.

In order to reveal the molecular mechanism of the p.K660N and another recently described somatic breast cancer mutation in FGFR2, p.R203C [32], expression vectors that direct the synthesis of FGFR2-IIIb carrying the breast cancer mutations were constructed and tested for their biological activity following transient expression in HEK293 cells. The p.R203C mutation is located in the extracellular Ig domain 2 of the receptor and the arginine at this position is highly conserved between species and all four human FGFRs (Fig. 2). The tyrosine kinase activities of FGFR2-IIIb carrying breast cancer mutations were compared to those of wt FGFR2-IIIb, of a dominant-negative kinase-defective (KD) FGFR2 mutant (p.K508A), and of a Pfeiffer syndrome gain-of-function FGFR2 mutant (p.K642R; previously named p.K641R according to another reference sequence) as controls. Lysates from cells expressing wt FGFR2-IIIb or one of the various mutants were subjected to immunoprecipitation with anti-FGFR2 antibody followed by SDS-PAGE and immunoblotting with anti-p-Tyr antibody. The intrinsic tyrosine kinase activities in both mutant proteins (p.R203C and p.K660N) were strongly increased as compared to the tyrosine kinase activity of wt FGFR2-IIIb (Fig. 5A). Different degrees of tyrosine autophosphorylation were detected for all FGFR2-IIIb mutants with the p.K660N mutant showing the highest tyrosine kinase activity.

Moreover, cells expressing wt and mutant FGFR2-IIIb were stimulated with FGF1 and total lysates from unstimulated and FGF1-stimulated cells were analysed. Both breast cancer mutants led to an increased tyrosine phosphorylation of FRS2, a well-characterized FGFR2 substrate, as revealed by immunoblotting with anti-p-FRS2 antibody after SDS-PAGE. We also showed that MEK1/2 and STAT3 stimulation in response to FGF1 stimulation is increased in HEK293 cells expressing the FGFR2-IIIb breast cancer mutants. In addition, results indicated that the responses of downstream effectors are most likely ligand-independent.

On the basis of these experiments, we conclude that the identified somatic breast cancer mutations are of an activating nature resulting in altered FGF-signalling.

## Discussion

The aim of our study was to gain new insights into the role of private somatic mutations in *FGFR2* and *FGF10* in breast tumor development. Here, we identified a somatic heterozygous missense mutation in exon 14 of *FGFR2*, c.1980G>C, that is predicted to lead to a substitution of a highly conserved lysine in the tyrosine kinase domain at position 660 to asparagine (p.K660N). We could clearly show that FGFR2-IIIb is predominantly expressed in tumor tissue of sporadic breast cancer patients, strongly suggesting that the p.K660N mutation is primarily mediated by this isoform. This finding is important for functional analysis of FGFR2 breast cancer mutations. Moreover, there has already been strong evidence that the isoform IIIb and not isoform IIIc of FGFR2 is associated with breast cancer development, since *FGF10*, the main ligand of FGFR2-IIIb and not FGFR2-IIIc, is amplified in approximately 10% of breast cancers and genome-wide association studies identified variants near *FGF10* as a genetic risk factor for breast cancer susceptibility, possibly influencing *FGF10* expression [6]. In order to unravel the molecular mechanism of the p.K660N and another recently described somatic breast cancer mutation in FGFR2, p.R203C, we compared the tyrosine kinase activities of breast cancer mutants to that of wt FGFR2. Our results clearly show that the intrinsic tyrosine kinase activity of both breast cancer mutant proteins is strongly increased. Furthermore, both breast cancer mutations implicated an increased tyrosine phosphorylation of the critical FGFR2 substrate FRS2 and an increased MEK1/2 and STAT3 activation in response to FGF1 stimulation leading to an accelerated cell signaling. To our knowledge this is the first report of a functional analysis of somatic breast cancer mutations in FGFR2 providing evidence for the activating nature of identified mutations. We also performed MLPA analysis to detect copy number variations in *FGFR2* and *FGF10* in sporadic breast cancer patients but did not identify any copy number changes in either of the two genes. We therefore speculate that those types of mutations do not play any important role in breast tumor development.

Signalling pathways activated by FGFs and FGFRs have been identified in multicellular organisms from *Caenorhabditis elegans* to vertebrates. It is now well established that the FGFR family of receptor tyrosine kinases and their numerous ligands play crucial roles in many developmental and physiological processes and that a variety of diseases are caused by aberrant signalling induced by FGFs or FGFRs [10], [11]. In humans, both loss- and gain-of-function heterozygous mutations have been described. Several human skeletal dysplasias are caused by gain-of-function mutations in *FGFR1*, *FGFR2* and *FGFR3*. Activating mutations located in the extracellular ligand binding domain were found in FGFR1 and FGFR2 associated with Pfeiffer, Crouzon, Jackson-Weiss and Apert syndromes and in the FGFR2 kinase domain associated with Pfeiffer and Crouzon syndromes. Likewise, activating mutations in FGFR3 are found in the transmembrane and the tyrosine kinase domains e.g. in achondroplasia, thanatophoric dysplasia type I (TDI) and type II (TDII) [12], [33], [34], [35]. Several of the FGFRs have been implicated in cancer through chromosomal translocations, activating mutations and aberrant splicing. Analyses of mutations from protein kinase screens performed in several cancer types further implicate the FGF signalling pathway in tumorigenesis [5]. The molecular consequences of these mutations are complex and may affect receptor activity, receptor stability, as well as receptor localization.

Interestingly, the novel p.K660N mutation identified in our study has also been found in endometrial carcinoma [36]. Additionally, germline mutations at the paralogous position have been identified in *FGFR3* associated with three different skeletal syndromes – TDII (p.K650E), severe achondroplasia with developmental delay and aconthosis nigricans (SADDAN) syndrome, and TDI (both due to p.K650M) [37], [38], [39]. The lysine at position 660 in FGFR2 and the equivalent lysine at position 650 in FGFR3 are strongly conserved between species and all four human FGFRs and located within the activation loop of the FGFR2 and FGFR3 tyrosine kinase domain, indicating that these sites are of functional importance. Amino acid substitutions at this position have been shown to have a dramatic effect on the constitutive activation of FGFR3-mediated receptor autophosphorylation [40], [41]. The activation loop of FGFRs is thought to block substrate binding until FGF binding and receptor dimerization alter its conformation, permitting both autophosphorylation of the receptor and phosphorylation of intermediate signalling molecules [42]. It has been demonstrated that the lysine 650 plays a critical role in stabilizing the FGFR3 activation loop in an inactive conformation, since different mutations of this residue constitutively activate the tyrosine kinase to varying degrees whereas mutations of adjacent residues have little effect. In addition, mutations of this residue may preclude the need for receptor dimerization at the cell membrane to activate the tyrosine kinase [41], [43]. Consistent with this study, we also demonstrated that the p.K660N mutation in FGFR2 is of an activating nature and effects likely ligand-independent autophosphorylation of the receptor.

In 2005, *Stephens et al* examined the coding sequence of 518 protein kinases, ∼1,3 Mb of DNA per sample, in 25 breast cancers. This was the first mutational screen of the full coding sequence of all protein kinases in cancer. A few tumors had numerous somatic mutations with distinctive patterns indicative of either a mutator phenotype or a past exposure. In one breast cancer, *Stephens et al* identified at least a single somatic heterozygous missense mutation in FGFR that is predicted to lead to a substitution of a highly conserved arginine in the tyrosine kinase domain at position 203 to cysteine (c.607C>T, p.R203C). Their results indicated that there is no commonly point-mutated and activated protein kinase gene in breast cancer. They suggested that approximately two thirds of the observed mutations are probably passenger mutations that are not subject to selection and, therefore, occur at a similar frequency throughout the genome [32]. *Stephens et al* did not perform any functional analysis of identified mutations in their study. We now show that the p.R203C mutation in FGFR2 leads to an increased intrinsic tyrosine kinase activity compared to that of wt FGFR2. However, the activating effect of the p.R203C mutation is lower compared to the p.K660N mutation. The arginine at position 203 is located in the extracellular Ig domain 2 of the receptor, which is responsible for heparin and ligand binding. Probably structural molecular changes arising from this amino acid substitution cause an increased ligand affinity of the receptor or ligand-independent activation. The exact mechanism needs to be elucidated.

In summary, we showed that identified somatic missense mutations in *FGFR2* are of an activating nature resulting in altered cell signalling. Our study provides further evidence for the implication of FGFR2 in tumor development. We suggest that the p.K660N and p.R203C mutations likely have driven clonal amplification of mutant cells.

In 2010, *Goriely and Wilkie* highlighted that paternal age effect (PAE) mutations are an emerging mechanism contributing to the introduction of new disease alleles into the population. PAE mutations mostly encode mutant proteins with gain-of-function properties, are of near-exclusive paternal origin, occur at elevated paternal ages and have an apparent germline mutation rate [44]. Interestingly, PAE mutations have been reported in various craniosynostosis syndromes caused by mutations in *FGFR2* and *FGFR3*. It has been shown that PAE mutations are enriched over time because they confer a selective advantage to the mutant spermatogonial stem cells, leading to their clonal expansion. This process has also been linked to the origin of testicular tumors and it has been suggested that the mechanisms involved in selfish selection are similar to those described for tumorigenesis [14], [45].

Furthermore, we propose that the phenotypic outcome of impaired FGF signalling caused by breast cancer mutations in the *FGFR2* gene is further modified by genetic and environmental factors which remain to be discovered. Both mutations influence tumor development by increasing, enhancing and accelerating tumor growth.

## Supporting Information

Table S1 **Primer for** ***FGFR2*** **amplification.**
(DOC)

Table S2 **Allele frequencies of detected SNPs.**
(DOC)
