# Supplementary material for: Activating Somatic FGFR2 Mutations in Breast Cancer
Source: PLoS One. 2013 Mar 20;8(3):e60264. doi: 10.1371/journal.pone.0060264 (PMC3603931; doi:10.1371/journal.pone.0060264)
Supplement: Table S1 — Primer for FGFR2 amplification. (DOC) [file pone.0060264.s001.doc]

| Exon | Direction | Primer Sequence | PCR Annealing Temp. |
| --- | --- | --- | --- |
| 7 | F | GTG GAC AGC CAA TAA CCT | Touchdown 66 - 56 |
|  | R | ATC AAC ACT GGC ACA ATG |  |
| 8 (IIIb) | F | CCA GCT AAT AAT TCC AAG | 51 |
|  | R | GCC TAA ACA AGA TCA ACC |  |
| 8 (IIIc) | F | AGT CGG AAT CTC CCA GTG | Touchdown 66 - 56 |
|  | R | GTC CAT TCT ATC GCA ACA |  |
| 9 | F | ATT GTC ACT GTC TCC TTA | Touchdown 66 - 56 |
|  | R | CCC ATT GAT CCA AGC AAC |  |
| 12 | F | GGT TAA GAA TGC TTG GTC | Touchdown 66 - 56 |
|  | R | ATC CAA AGT CAC CTG CAC |  |
| 13 | F | CCA AGG GCT TAT TTA TCA | 51 |
|  | R | AAC TCA AAT GGG AAT AAC |  |
| 14 | F | CTG GCG GTG TTT TGA AAT TAG | 51,4 |
|  | R | CCT AGC GGT TGC TGA TTA TTC |  |
| 15 | F | GAA GAA AGG CCG TCA ATG | 51 |
|  | R | AAA GAA GGA AGA AAG GT |  |
