# Supplementary material for: Activating Somatic FGFR2 Mutations in Breast Cancer
Source: PLoS One. 2013 Mar 20;8(3):e60264. doi: 10.1371/journal.pone.0060264 (PMC3603931; doi:10.1371/journal.pone.0060264)
Supplement: Table S2 — Allele frequencies of detected SNPs. (DOC) [file pone.0060264.s002.doc]

| **SNP** | **localization** | **∑ patients** | **11** | **12** | **22** | **1** | **2** | **HapMap** | |
| --- | --- | --- | --- | --- | --- | --- | --- | --- | --- |
|  |  |  |  |  |  |  |  | **1** | **2** |
| rs2981448 | intron 7 | 33 | 32 (0,970) | 1 (0,030) | 0 (0) | 0,985 | 0,015 | 1 | 0 |
| rs2071616 | intron 7 | 33 | 16 (0,485) | 12 (0,364) | 5 (0,152) | 0,667 | 0,334 | 0,667 | 0,333 |
| rs3135802 | intron 17 | 167 | 165 (0,988) | 2 (0,012) | 0 (0) | 0,994 | 0,006 | 0,992 | 0,008 |
| rs2981460 | intron 16 | 145 | 86 (0,593) | 47 (0,324) | 12 (0,083) | 0,755 | 0,245 | 0,714 | 0,286 |
| rs2981461 | intron 16 | 145 | 59 (0,407) | 58 (0,400) | 28 (0,193) | 0,607 | 0,393 | 0,560 | 0,440 |
| rs1649199 | intron 16 | 145 | 110 (0,759) | 33 (0,228) | 2 (0,014) | 0,930 | 0,128 | 0,817 | 0,183 |
